# How do fathers’ educational level contribute to children’s school problems? Overparenting and children’s gender and surgency in a moderated mediation model

**DOI:** 10.3389/fpsyg.2024.1405389

**Published:** 2024-10-21

**Authors:** Rosa María Ruiz-Ortiz, Rosario Carreras, Nora del Puerto-Golzarri, José Manuel Muñoz

**Affiliations:** ^1^Department of Psychology, University of Cádiz, Cádiz, Spain; ^2^Department of Basic Psychological Processes and Their Development, University of País Vasco, San Sebastián, Spain

**Keywords:** moderated mediation model, father’s educational level, school problems, overparenting, child’s gender, surgency, shyness

## Abstract

This study aims to investigate (a) the mediating role of overparenting between father’s educational level and children’s school problems, and (b) the joint moderating role of children’s gender and surgency in the indirect relationship between father’s educational level and school problems. Participants were 203 school children, 96 boys (47.3%) and 107 girls (52.7%), aged 7–8 years (M = 92.42 months, SD = 3.52). Fathers reported their educational level, age and employment status and their children’s gender and number of siblings, as well as their overparenting behaviors by Anticipatory Problem Solving (APS) scale. Teachers informed children’s school problems by the Behavior Assessment System for Children (BASC T-2). Parents together informed their children’s surgency levels by a subscale of Children’s Behavior Questionnaire (CBQ). Results showed that, in girls, the father’s educational level was negatively related to the child’s school problems via overparenting behaviors, controlling the number of siblings and father’s age and employment status. However, among boys, fathers’ overparenting protect their shy sons from the risk of a low educational level for school problems. These findings highlight the relevance of considering the gender and surgency to a better understanding of the effects of contextual factors on children’s outcomes.

## 1 Introduction

During middle childhood, adaptation to school and academic success becomes a key developmental task for children (Walker and Graham, 2021). Children who start elementary education with regulation and attention skills are more engaged in learning and have a better school adjustment (Rabiner et al., 2016; Trentacosta and Izard, 2007). By contrast, school problems during the first years of middle childhood (academic difficulties related to motivation, attention and learning) are strong predictors of negative outcomes in adolescence such as later school failure, violent behaviors, mental health problems and maladaptive trajectories (Cunha and Heckman, 2009; Hughes, 2011; Kellam et al., 2008; Perren and Alsaker, 2006; Petras et al., 2008; Rojas Andrade and Leiva Bahamondes, 2015; Schwartz et al., 2008; Vargas et al., 2019). Since the early identification of children at risk for school problems can help prevent these negative outcomes, we address what determines the school problems in children 7 and 8 years old.

School-age children with considerable support from their parents have consistently shown a more significant behavioral, cognitive, and socio-affective involvement in the learning and higher level of academic skills (Careemdeen et al., 2020; Felizardo et al., 2016). Yet to date, findings about the parent’s impact on children’s outcomes have been overwhelmingly focused on mothers, who have played a priority role in childcare around the world (Bornstein and Putnick, 2016; Downer et al., 2008). In recent decades, given that the number of working mothers has increased and there have also been social changes around gender roles, there is increasing research on the fathers’ role in early child development (Cabrera et al., 2007; Cowan et al., 2009; Marsiglio et al., 2000; Panter-Brick et al., 2014). In relation to the influence of fathers on children’s academic skills, the results of most studies have shown that it is positive and statistically significant, although there has been very little research conducted with middle childhood samples (Rollè et al., 2019). Considering the need for more studies about the role of fathers in child development in this age period, we examine the contribution of paternal factors to children’s school problems.

Based on Bronfenbrenner’s ecological theory (Bronfenbrenner, 1979), for a better understanding of paternal influences on children’s school skills and academic success, it is necessary to take into account both distal and proximal factors. Regarding distal factors, many studies suggest that the educational level of parents is a key factor which affects the cognitive and non-cognitive outcomes of their children (e.g., Wang et al., 2020). In relation to proximal factors, research has highlighted the relations between paternal involvement during early and middle childhood and school performance (Fan and Chen, 2001; Jeynes, 2007; Lankinen et al., 2020; Rempel et al., 2020; Rollè et al., 2019). However, what happens when the involvement is too intensive? Overparenting is a term used to describe a parenting style characterized by excessive involvement of parents, who give too much advice, provide sometimes even unnecessary assistance and solve problems for their children (Segrin et al., 2012). Some studies have found that, among the parental behaviors that characterize overparenting, the one that best predict their children’s psychosocial development is the anticipation and resolution of the problems their kids may encounter in their lives, so scholars suggest this behavior might be the most appropriate for measuring overparenting (Scharf and Rousseau, 2017; Schiffrin et al., 2015). Following these authors, we conceptualize overparenting as a form of parenting that is used by parents whose concern for the success, happiness, and wellbeing of their children leads them to solve problems for their kids, perhaps before they even develop, removing any perceived obstacles to those positive outcomes. Therefore, this behavior of anticipating the problems of their children [Anticipatory Problems Solving (APS); Segrin et al., 2012], as a relevant or main feature of overparenting, is one of the paternal factors considered in this study. With all this in mind, we focus on the relations between two fathers’ factors (educational level as distal factor and APS as proximal factor) and their children’s school problems in middle childhood.

## 2 Fathers’ educational level and children’s school problems

Several studies have considered the effects of fathers’ education on children’s academic outcomes (Cabrera et al., 2007; Malin et al., 2012; Michiels et al., 2010; Newland et al., 2013; Sarkadi et al., 2008). Among them, D’Urso and Symonds (2022) concluded that father’s educational level was one of the best predictors of pupils’ academic performance. Others have found, in a sample of elementary students, that the effect of the fathers’ educational level on their children’s mathematics attainment was positive and significant, while this effect was not found with respect to literary skills (Park and Hannum, 2001).

It is possible that the link between fathers’ education and children’s school outcomes may be affected by some other intervening factors. For example, a study carried out with Spanish samples of children and adolescents (Padilla-Moledo et al., 2016) found an association between father’s educational level and children’s academic performance but did not observe these associations among adolescents. These authors consider that as children grow and become adolescents, the influence of the parents’ educational level seems to decrease in favor of the influence of aspects related to school, peers, and family environment. Thus, based on previous research in middle childhood and considering, in most western societies, fathers emphasize the importance of academic achievement as an important developmental task for their school-aged children (McGrath and Repetti, 2000; Sabatier and Lannegrand-Willems, 2005), we hypothesized that father’s educational level is negatively related to elementary students’ school problems. We also consider that it is relevant to examine possible variables that may explain why a father’s educational level has a meaningful impact on their children’s school problems.

### 2.1 The mediating role of anticipatory problems solving

Despite that overparenting is a well-intended practice, is also generally understood that excessive protection of their children and solving their problems for them (APS) have an adverse impact on children’s outcomes (Luebbe et al., 2018; Reed et al., 2016; Segrin et al., 2015). Self-determination theory (Ryan and Deci, 2000) could help explain why these behaviors could be related to maladjustment. According to this theory, contexts that prevent the satisfaction of autonomy, competence and relatedness (three basic psychological needs) negatively influence on psychological and behavioral development. Parents that provide excessive assistance and anticipatory problem solving implicitly convey negative feedback to children, suggesting that they are not competent and unable to tackle problems on their own. Consequently, these implicit messages would undermine children’s sense of competence and control (Hong and Cui, 2020).

From an empirical point of view, much of the overparenting research has been conducted with students in high school or college, showing that this parenting behavior can lead to high levels of narcissism, dependence on others and depression and low levels of self-efficacy in the students (Bradley-Geist and Olson-Buchanan, 2014; Odenweller et al., 2014; Schiffrin et al., 2015; Segrin et al., 2013). Nevertheless, these same parenting behaviors may be developmentally appropriate in other developmental periods. For example, in infants to 3–4 years old, benefits of high levels of parental assistance to their children in anticipating and solving problems include cognitive, physical and socioemotional development (Schiffrin et al., 2015). However, the influence is detrimental to children’s development when parents are not able to reduce these monitoring behaviors as their children grow. Although there is also little research on the impact of overparenting on children in elementary schools, some studies have shown that overprotective parents avoid the experience of failure to their children, complicating the development of their problem-solving and task-management skills on their own (Fearon and Roisman, 2017; Randall et al., 2015) as well as their independence and ability to choose (Liss and Schiffrin, 2014). This may become problematic for children’s emotional development (Hussain et al., 2021; Perry et al., 2018), and inability to socialize and deal with failure, and even developmental delays (Padilla-Walker et al., 2021; Valente et al., 2017). It is not known when anticipatory problem solving could begin to have negative effects on children’s development. Therefore, more research is needed to improve our understanding of the impact of these parenting behaviors on elementary school children (Turner et al., 2022).

On the other hand, parent educational attainment is an important background factor and often linked to parenting practices (Azad et al., 2014; Hoff and Laursen, 2019). Popularly, excessive involvement in helping their children solve their problems tends to be used more frequently by families with a higher socioeconomic level, given the belief that these parents have more time to focus on raising their children and their success is assumed as their own (Segrin et al., 2013; van Ingen et al., 2015). Thus, these overparenting behaviors have been associated with higher parents’ educational level in the media (Gibbs, 2009). However, the empirical evidence available in this regard is scarce; rather, the few studies conducted show that parents with a high level of education were less likely to use overprotective behaviors (e.g., Sleddens et al., 2014).

More research is needed to better understand the associations between sociodemographic characteristics, overparenting and children’s outcomes in elementary school (Arlinghaus et al., 2023). Considering findings of previous research, we hypothesized that Anticipatory Problem Solving by fathers is not only associated with the school problems of their children, but also mediates the relations between fathers’ educational level and children’s school problems. Understanding the role that anticipatory problem solving plays in promoting children’s school success could have relevant implications for parenting and education practices.

### 2.2 The moderating role of children’s gender and surgency

The Environmental Sensitivity Theory (Greven et al., 2019; Pluess, 2015) is known as an evolutionary-developmental theory, which explains that humans differ in their sensitivity to contextual factors. Several studies conducted under the umbrella of Environmental Sensitivity Theory have reported gender differences in favor of females in Sensory Processing Sensitivity (Benham, 2006; Chacón et al., 2021; Konrad and Herzberg, 2017). Sensory Processing Sensitivity has been defined as a genetic-based personality trait characterized by an ability to respond to external factors and process sensory information more deeply (Assary et al., 2021; Pluess, 2015). This personality trait involves a higher sensitivity to various stimuli, such as environmental influences from groups, families, schools, etc. (Aron et al., 2012; Aron and Aron, 1997; Jagiellowicz et al., 2011; Pluess, 2015).

Moreover, there are other reasons for testing for differences depending on a child’s gender in the associations of contextual paternal factors and children’s outcomes. Firstly, since the 90s, several studies have shown, in countries committed to equal opportunities, gender differences in favor of girls in diverse measures related to school performance (Epstein et al., 1998; Freudenthaler et al., 2008; Steinmayr and Spinath, 2008; Wong et al., 2002). Secondly, there is also some research exploring the relations between child gender and factors commonly associated with overparenting as parental monitoring or involvement that suggests child gender does influence levels of these parental behaviors (Padilla-Walker and Nelson, 2012) with female children generally reporting greater rates (Bradley-Geist and Olson-Buchanan, 2014; Klevens and Hall, 2014). Thirdly, several studies testing for gender differences in the relation between parenting and child outcomes suggest that girls appear to be more sensitive to parenting practices than boys (e.g., Lewis-Morrarty et al., 2015), hence parenting may be a more intense predictor of child’s outcomes for girls than for boys (Leung, 2020).

There are also some studies, carried out on adolescents or emerging adults, that have analyzed the moderating role of gender in the association of overparenting with child’s outcomes. Although, some of these researches have not found gender differences on this association (e.g., Darlow et al., 2017; Scharf et al., 2017), most of them have suggested that overparenting seems to increase risk of maladjustment concretely for girls (Barton and Kirtley, 2012; Janssens et al., 2009; Kouros et al., 2017; Patock-Peckham and Morgan-Lopez, 2006). In the present study, we analyze the moderating role of the child’s gender in the effects of father’s educational level on APS and school problems of children during childhood. Although we have not found previous studies in this period of age, we hypothesize, based on the results discussed, that these effects will be stronger among girls than among boys.

On the other hand, we also tested surgency as an additional moderator. Surgency (extraversion) is defined as the level of arousal and positive affect regarding an activity or change, as well as a low level of behavioral inhibition or shyness (Rothbart, 2007). Some research has showed that higher levels of surgency were predictive of higher levels of motivation for school (Chen and Zhang, 2011) and higher levels of academic success in middle childhood (Bramlett et al., 2000; Colom et al., 2007; Rudasill et al., 2010). In contrast, shyness (an indicator of low surgency) has been sometimes negatively associated with academic outcome (Hughes and Coplan, 2010; Zhang et al., 2017). Thus, for highly shy children, even going to school can be a challenge, causing them academic difficulties due to their low levels of emotional and behavioral engagement, low levels in language skills, low school liking and less participation in teacher-child interactions (Cameron, 2009; Eggum-Wilkens et al., 2014; Rudasill, 2011; Valiente et al., 2012).

Moreover, research carried out in the framework of Environmental Sensitivity Theory has found that introversion (or low surgency) is related to higher levels of sensitivity to the environment, such as parental influences (Asscher et al., 2016; Hentges et al., 2015; Pluess et al., 2010). For instance, parents’ negative affect predicted childhood anxiety problems for highly shy children (an indicator of low surgency), but not for other children (Lindhout et al., 2009). However, other studies have found that children with high levels of surgency seem to be more sensitive to the effects of parenting; for example, del Puerto-Golzarri et al. (2022) found that boys with high levels of surgency whose fathers used an authoritarian parenting style showed more reactive aggression. We have only found a study that examines whether surgency would moderate the link between APS and child’s behavior problems, such as aggression (Ruiz-Ortiz et al., 2023). This study showed that only among children with high levels of surgency, overprotection reduced levels of child’s aggression.

Lastly, it seems there is a relation between surgency and gender. Else-Quest et al. (2006) found that surgency showed a difference, favoring boys, in particular with boys scoring higher than girls. Other studies have found gender differences in the components or surgency. Thus, boys are higher on activity level and high-intensity pleasure (Gaias et al., 2012; Muris and Meesters, 2009). Moreover, findings about shyness have suggested that this temperament trait may be less socially acceptable for boys than for girls, even parents seem to react more negatively to shy behaviors in boys as compared to girls (e.g., Bosacki, 2008; Rubin and Coplan, 2004). For all these reasons, we are interested in including surgency as an additional moderator in our study and the previous findings lead us to hypothesize that we will find triple interactive effects of paternal factors with child’s gender and surgency on children’s school problems. Given the lack of prior specific research about the possible direction of this moderation, this research aim was exploratory.

## 3 Hypotheses and overview of study

The present study focuses on the relationship among fathers’ educational levels and children’s school problems, as well as the mediating role of overparenting behaviors and the moderating role of children’s gender and surgency. Based on previous findings, the present study proposes the following hypotheses:

Hypothesis 1 (H1): Fathers’ educational level would be negatively related to children’s school problems.

Hypothesis 2 (H2): APS would be positively related to children’s school problems.

Hypothesis 3 (H3): APS would mediate the relationship between fathers’ educational level and children’s school problems.

Hypothesis 4 (H4): Gender would buffer the effects of fathers’ educational level and APS on children’s school problems, such that among girls the negative relation between father’s educational levels on children’s school problems and the positive relation between APS on children’s school problems would be stronger than among boys.

Hypothesis 5 (H5): Child’s gender would moderate the relationship between fathers’ educational level and children’s school problems via APS, such that the indirect effect between fathers’ educational level and children’s school problems via APS is stronger for girls.

Hypothesis 6 (H6): Child’s surgency would act as an additional of moderator of the moderated mediation model hypothesized in H5, that is, there would be a triple interactive and significant effect of each paternal factor (father’s educational level and APS) with child’s gender and surgency on school problems.

Hypothesis 7 (H7): Child’s surgency would moderate the effect of gender in the relation between fathers’ educational level and children’s school problems via APS, that is, there would be differences depending on child’s gender in moderation that the child’s surgency levels exerts in the indirect effect of father’s educational level on child’s school problems via APS.

**Figure 1:**
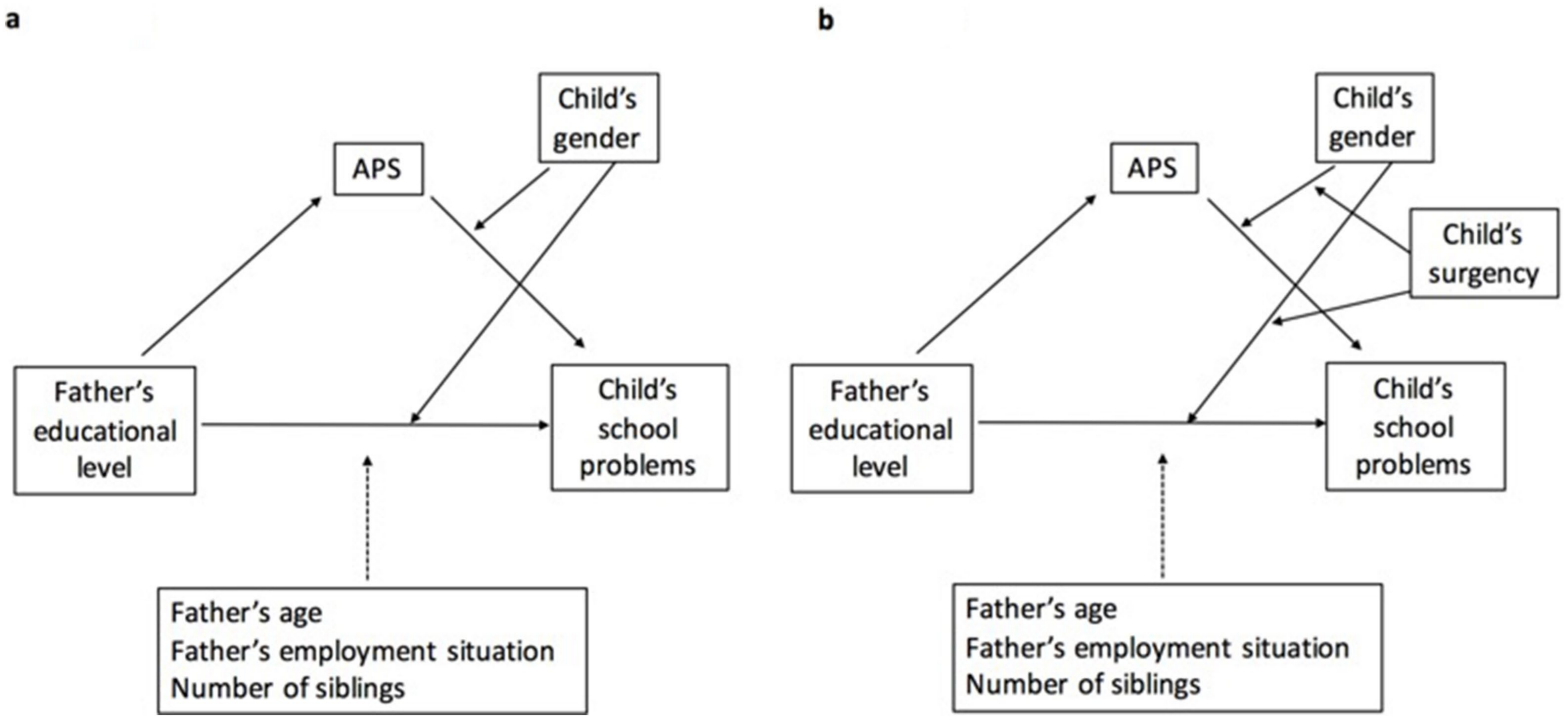
The proposed conceptual model. (a) Gender moderating the mediation model. (b) Gender and surgency moderating the mediation model.

In summary, the main aim of this study was to discuss the mediating mechanism of APS and the moderating effects of gender in the relation between the father’s educational level and children’s school problems (Figure 1a). An additional goal was to analyze whether these mediating and moderating effects change depending on the surgency levels (Figure 1b).

## 4 Materials and methods

### 4.1 Participants and procedure

The sample was selected by age and sex, according to geographical localization. Five governmental schools in southern Spain (Cádiz) agreed to participate in the study including 14 classrooms with 335 students. A comprehensive verbal and written description of the purpose of the study was given to the children’s parents and teachers. Participation in the study was voluntary and anonymous and that the study adhered to all ethical guidelines. Written consents from 252 families were obtained. Once the families returned the completed questionnaires and the data was coded, a final sample of 203 schoolchildren, 96 boys (47.3%) and 107 girls (52.7%), aged 7–8 years (M = 92.42 months, SD = 3.52) participated in the study. Fathers’ average ages were 37.59 years (SD = 7.97). The 26.6% of fathers completed primary education, 10.8% secondary education, 47.7% high school or higher vocational training studies and 14.8% university studies.

In order to avoid common method biases, sample data was collected at three time points. Data about fathers’ educational level was obtained at T1 (first term or school year) by an *ad hoc* sociodemographic questionnaire. During the second term of school year (T2), fathers filled the Overparenting Scale, concretely the Anticipatory Problems Solving subscale (Segrin et al., 2012) and fathers and mothers jointly informed about children’s surgency by the Children’s Behavior Questionnaire (CBQ; Rothbart et al., 2001). In the third term of school year (T3), teachers reported information about their students’ school problems completing the Behavior Assessment System for Children (BASC T-2, Reynolds and Kamphaus, 2004). Given common method bias basically occurs when all data are obtained from the same sources or informants, we tried to prevent it by implementing a procedural remedy, as recommended by Podsakoff et al. (2003). Thus, we collected the measures of the variables from key but different informants (APS by father; School problems by teacher; child’s surgency and demographic variables by father and mother jointly in different time). This procedure can prevent the informant’s mentality from biasing the observed relationship between the variables, thus eliminating the effects of implicit theories, social desirability, or evaluator moods (Podsakoff et al., 2003).

### 4.2 Measures

#### 4.2.1 School problems

Teachers reported information about students’ school problems (outcome variable) completing the Behavior Assessment System for Children (BASC T-2, Reynolds and Kamphaus, 2004; Spanish adaptation, González et al., 2004). This instrument contains 149 items scored on a Likert-type scale ranging from (A) “never” a (D) “almost always.” In this study, only the School Problems subscale that includes academic difficulties related to motivation, attention, learning and cognition (e.g., “He/she makes mistakes because he/she is not attending”) was used (*α* de Cronbach 0.93). The School Problems composite for the BASC-3 is derived from the Attention Problems and Learning Problems subscales. Items pertaining to the Attention Problems subscale measure problems of distractibility and the ability to pay attention. The Learning Problems subscale is focused on critical thinking skills, completion of assignments, math, reading, and spelling.

### 4.3 Father’s educational level

Fathers reported their educational level through the *ad hoc* sociodemographic questionnaire, based on Antolín et al. (2009). Fathers’ education levels (predictor variable) were coded into five categories: (1) primary school, (2) secondary school, (3) high school, (4) higher vocational school, and (5) university degree or higher. Fathers indicated their highest level of education in the five points scale. Fathers’ education levels were coded from 1 to 5. The larger the numbers are, the higher the education level is.

#### 4.3.1 Anticipatory problem solving

The anticipation and resolution that parents do of their children’s problems (mediating variable) was assessed using the Anticipatory Problem Solving (APS) subscale of the Overparenting Scale (Segrin et al., 2012). The APS subscale was selected because it better operationalized the behaviors that could occur as a result of overprotection. Other studies have also suggested that this subscale is appropriate to measure overparenting (Scharf and Rousseau, 2017; Schiffrin et al., 2015). This subscale provides information about the degree to which fathers solve their children’s problems offering them assistance or removing obstacles for them. The subscale includes 12 items measured on a scale of “strongly disagree” (1) to “strongly agree” (5) (e.g., “I try to help my child steer clear of any troubles that s/he might encounter in the world”). The reliability of this measure was 0.87 in the original study and 0.82 in the current study.

#### 4.3.2 Child’s gender

This variable (moderating variable) was coded as girl (1) and boy (0) for the current study.

#### 4.3.3 Children’s surgency

Children’s surgency (moderating variable) was measured using the surgency dimension of the short version of the Children’s Behavior Questionnaire (CBQ; Putnam and Rothbart, 2006) completed by parents. This dimension questionnaire consists of 12 items scored on a Likert-type scale ranging from “extremely false” (1) to “extremely true” (7) (e.g., “He/she seems always in a big hurry to get from one place to another,” “He/she likes going down high slides or other adventurous activities”). It includes different subscales (Cronbach’s α = 0.71): activity level, high intensity pleasure, impulsivity, and shyness (inversed).

### 4.4 Covariates

Due to their documented association with school problems, father’s employment status, number of siblings and father’s age were treated as covariates. It seems be clear that parents’ employment status plays a significant role on students’ academic performance, either because of a better employment status provide necessary facilities needed for the enhancement of their children education (Suleman et al., 2012) or because of parental employment affects the time spent with their children (Schildberg-Hörisch, 2016). Furthermore, some studies have pointed out that educational performance is negatively influenced by a high number of siblings (Baert et al., 2022; Biblarz and Raftery, 1999; Björklund and Sundström, 2006; Garasky, 1995; Ghysels and Van Vlasselaer, 2008). On the other hand, parent age and the number of siblings are likely to influence interactively on children’s performance. Hence, we control for each factor separately.

The information about age, fathers’ employment status and number of siblings was reported by fathers through the *ad hoc* sociodemographic questionnaire. Father’s age and number of siblings were used as continuous variables. Fathers’ occupation was coded into five categories: (1) active, (2) unemployed with allowance, (3) unemployed without allowance, (4) retired and (5) others.

### 4.5 Statistical analysis

The study used SPSS 21 to carry out descriptive statistics and bivariate correlation analyses in order to test H1 y H2. The other hypotheses were tested using the PROCESS macro for SPSS (Hayes, 2018), using bias-corrected bootstrapping with 5,000 resamples to account for the non-normal distribution in the outcome variables (MacKinnon et al., 2004) and to generate confidence intervals (Preacher and Hayes, 2008). Specifically, in the current study models 4, 15, and 19 were used following Hayes’s recommendations (Hayes, 2018). Concretely, we used Model 4 and calculated 5,000 bootstrapped samples to estimate the 95% bias corrected and accelerated confidence intervals of the indirect effect to study direct and indirect effects of father’s educational level and APS on children’s school problems (H3). A mediation test is significant when the lower and the upper bounds of the bootstrap confidence intervals of the indirect effect between the predictor and the outcome do not include zero (Hayes, 2018). Instead of the traditional Sobel test, PROCESS model bootstrap approach was used, because the bootstrap method has higher statistical power and makes more realistic assumptions about the sampling distribution of the indirect effect (Abu-Bader and Jones, 2021; MacKinnon et al., 2004).

Further to test H4 and H5, we used Model 15 with bias-corrected bootstrap confidence intervals (BC; 95% CI) based on 5,000 bootstrap resamples. Model 15 was used to evaluate the moderation effect of children’s gender in both the direct and indirect effects of fathers’ educational level and APS on children’s school problems (Hayes, 2018). Lastly to test H6 and H7, Model 19 was used with bias-corrected bootstrap confidence intervals (BC; 95% CI) based on 5,000 bootstrap resamples. Model 19 was conducted to test three-way interactions, which is similar to model 15, but with a moderator influencing the indirect path by the mediator that depends on a second moderator (a moderated moderated mediation). This model was used to test if the moderated effect by child’s gender for father’s educational level on child’s school problems via APS would be at the same time moderated by child’s surgency (Hayes, 2018). Significant interactions were probed using a traditional simple slope test, the pick-a-point approach (Aiken et al., 1991), and the Johnson-Neyman technique for regions of significance (Hayes and Matthes, 2009; Preacher et al., 2006), which identifies the regions of significance of an association when the moderator is a continuous variable (Hayes, 2018). Since regression analyses included interaction terms, continuous variables that constitute an interaction product were mean centered to minimize multicollinearity issues (Field, 2013).

## 5 Results

### 5.1 Preliminary analyses

In the study, to determine the correlations between the variables, bivariate correlation analysis was applied. Table 1 provides the correlations for all variables. As the result of the analysis, between father’s educational level and APS and also between father’s educational level and child’s school problems, statistically significant negative correlations were found. In addition, a statistically significant positive correlation was detected between APS and child’s school problems. Thus, H1 and H2 were supported. The moderating variables (child’s gender and surgency) did not show a significant relationship with any other variable. Lastly, father’s age showed a statistically significant positive correlation with father’s educational level and negative with the child’s school problems. Father’s employment status was negatively associated with father’s educational level (note that regarding educational level, the larger the numbers are, the higher the education level is, and regarding employment status, the larger the numbers are, the less socially recognized employment status is).

**Table 1:** Descriptive statistics and correlations for all variables.

Variables	1	2	3	4	5	6	7	8
Child’s SP	–							
Father’s EL	−0.142*	–						
APS	0.172*	−0.210**	–					
Child’s gender	−0.112	−0.043	−0.059	–				
Surgency	0.095	0.061	0.011	−0.103	–			
Father’s age	−0.159*	0.170*	0.014	−0.095	−0.007	–		
Father’s ES	−0.046	−0.186**	0.069	−0.100	0.021	−0.019	–	
Number of siblings	0.110	0.048	−0.124	−0.084	0.103	0.014	0.013	–
*M*	47.059	2.833	0.000	0.530	0.000	40.414	1.458	0.941
*SD*	10.034	1.386	1.000	0.500	1.000	4.710	0.891	0.665

*N* = 203. Gender was dummy coded such that 0 = Boy and 1 = Girl. APS and Surgency variables were standardized. Child’s SP, Child’s school problems; Father’s EL, Father’s educational level; Father’s ES, Father’s employment situation. *p < 0.05, **p < 0.01.

### 5.2 Mediating analysis

Following the procedures outlined by Preacher et al. (2007), we used PROCESS macro in the SPSS software by selecting Model 4 (Hayes, 2018) to address H3, which stated the effect of father’s educational level on child’s school problems is mediated by APS. The analyses included father’s age and employment status and number of siblings as control variables. The results of the mediation analysis are presented in Table 2. As can be seen in Table 2, firstly the findings revealed that between father’s educational level and child’s school problems, after controlling for father’s age and employment status and number of siblings, there was a tendency toward statistical signification for negative association (b = −0.978, *p* = 0.060). Secondly, the results also indicated that father’s educational level was negatively associated with APS (b = −0.144, *p* = 0.006). Thirdly, APS was positively related to child’s school problems (b = −1.793, *p* = 0.015). Moreover, the residual direct association between father’s educational level and child’s school problems was not significant. Additionally, regarding control variables, father’s age negatively related to child’s school problems (b = −0.365, *p* = 0.016) and number of siblings had a positive and significant relation with child’s school problems (b = 2.333, *p* = 0.034), regardless of father’s educational level and APS. The bootstrapping analysis with 5,000 iterations revealed that APS mediated the relation between father’s educational level and child’s school problems. The 95% bias-corrected confidence interval for the indirect effect did not include zero (indirect effect = −0.258, SE = 0.142, 95% CI [−0.583, −0.033]) and the direct effect included zero (direct effect = −0.719, SE = 0.521, 95% CI [−1.748, 0.309]). Thus, H3 is supported. The mediation effect accounts for 26.40% of the total effect of father’s educational level on child’s school problems (Wen and Fan, 2015).

**Table 2:** Testing the mediating effect of APS in the relation between father’s educational level and child’s school problems.

	Outcome variables								
	School problems			APS			School problems		
Predictors	*b*	SE	*t*	*b*	SE	*t*	*b*	SE	*t*
Father’s EL	−0.978	0.517	−1.889	−0.144	0.052	−2.797**	−0.719	0.521	−1.380
APS							1.793	0.729	2.459*
Father’s age	−0.346	0.153	−2.269*	0.011	0.015	0.692	−0.365	0.151	−2.422*
Father’s ES	−0.817	0.799	−1.023	0.032	0.080	0.408	−0.875	0.788	−1.110
Number of siblings	2.027	1.097	1.848	−0.170	0.109	−1.560	2.333	1.089	2.141*
*R^2^*	0.071			0.060			0.100		
*F*	3.507**			2.942*			4.092**		

Each column is a regression model that predicts the criterion at the top of the column. Gender was dummy coded such that 0 = Boy and 1 = Girl. Results are based on 5,000 bootstrap samples. Father’s EL, Father’s educational level; Father’s ES, Father’s employment situation. *p < 0.05, **p < 0.01.

### 5.3 Moderated mediation analysis

**Figure 2:**
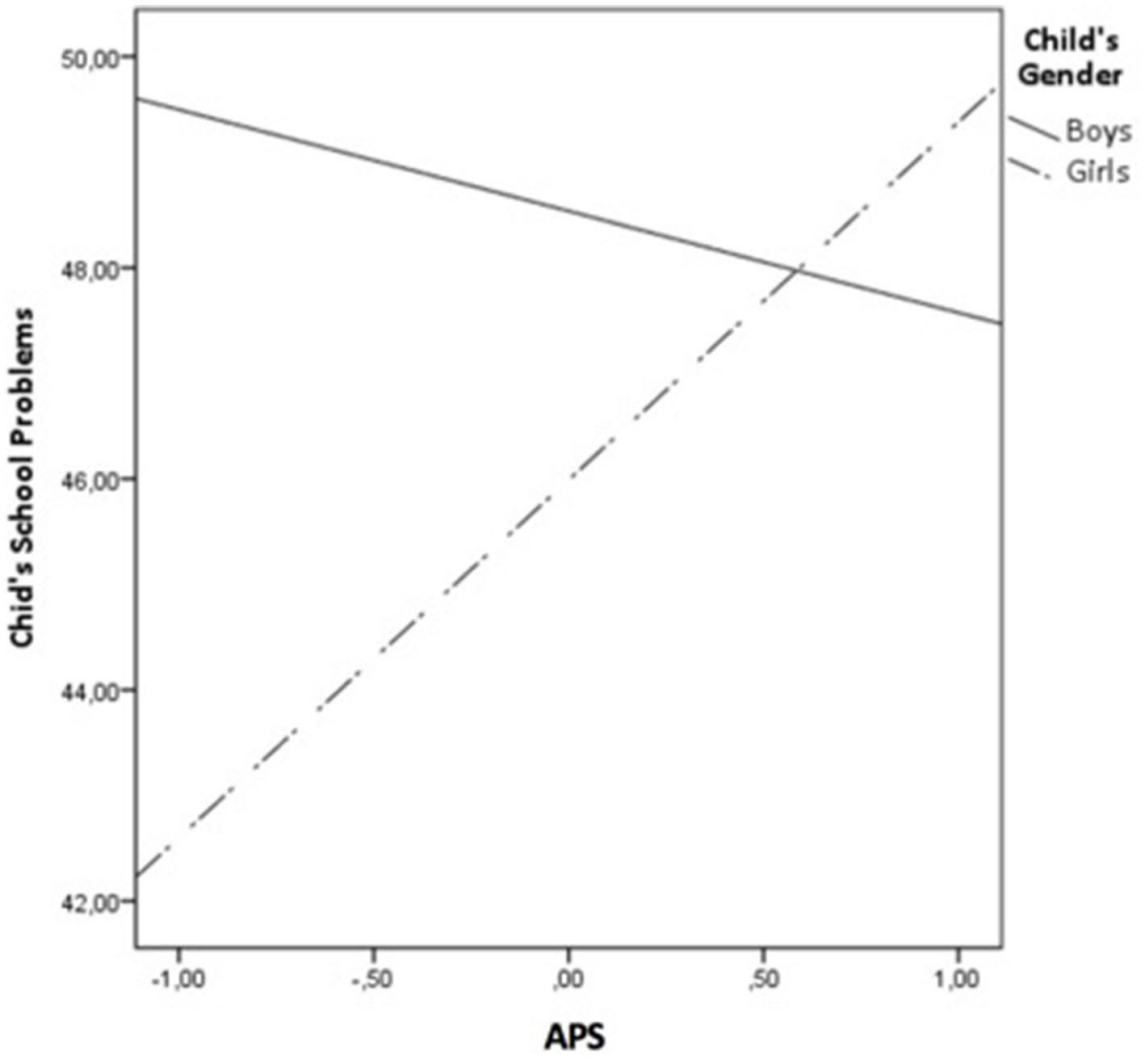
The moderation of gender in the relations between APS and child’s school problems.

In order to test H4 and H5, a moderated mediation model using PROCESS Model 15 (Hayes, 2018) was conducted to test whether gender had a moderating role in the direct effects of father’s educational level and APS on child’s school problems, as well as, in the indirect effect of father’s educational level on child’s school problems via APS, controlling for father’s age and employment status and number of siblings. The results in Table 3 showed that child’s gender moderated the relationship between APS and child’s school problems (b = 4.271, *p* = 0.004), whereas having no significant effect on the relation between father’s educational level and child’s school problems. In order to further analyze the APS × child’s gender interaction, a simple slope test was performed using the pick-a-point approach (Aiken et al., 1991). A significant positive relation between APS and child’s school problems was found among girls (b = 3.424, *p* = 0.000), while a non-significant negative relation was found among boys (b = −0.847, *p* = 0.450), as shown in Figure 2. Additionally, the index of moderated mediation was significant (index = −0.615, SE = 0.311, 95% CI [−1.333, −0.125], 5,000 bootstraps); only among girls, the indirect effect value of the father’s educational level on child’s school problems by APS was significant (boys, indirect effect = 0.122, SE = 0.182, 95% CI [−0.210, 0.530]; girls, indirect effect = −0.493, SE = 0.232, 95% CI [−1.027, −0.122]). Thus, H4 was only partially supported because child’s gender moderated the direct effect of APS on child’s school problems (only among girls, this direct effect was significant), but did not moderate the direct effect of father’s educational level on child’s school problems. H5 was supported because the indirect effect of fathers’ educational level on children’s school problems via APS was significantly different for boys than for girls.

**Table 3:** Testing the moderating effects of gender and in mediating effect of APS in the relation between father’s educational level and child’s school problems.

Predictors	Outcome variables					
	APS			School problems		
	*b*	SE	*t*	*b*	SE	*t*
Father’s EL	−0.144	0.052	−2.797**	−1.270	0.729	−1.743
APS				−0.847	1.117	−0.758
Gender				−2.987	1.395	−2.141*
Father’s EL × gender				0.737	0.992	0.743
APS × gender				4.271	1.451	2.943**
Father’s age	0.011	0.015	0.692	−0.362	0.148	−2.451*
Father’s ES	0.032	0.080	0.408	−0.902	0.775	−1.163
Number of siblings	−0.170	0.109	−1.560	1.786	1.073	1.665
*R^2^*	0.060			0.161		
*F*	2.942*			4.332***		

Each column is a regression model that predicts the criterion at the top of the column. Gender was dummy coded such that 0 = Boy and 1 = Girl. Results are based on 5,000 bootstrap samples. Father’s EL, Father’s educational level; Father’s ES, Father’s employment situation. *p < 0.05, **p < 0.01, ***p < 0.001.

### 5.4 Moderated moderated mediation analysis

In order to test H6 and H7, a moderated moderated mediation model using PROCESS Model 19 (Hayes, 2018), with child’s school problems as the outcome variable, father’s educational level as the predictor, APS as the mediating variable and child’s gender and surgency as the moderating variables, controlling for father’s age and employment status and number of siblings.

**Figure 3:**
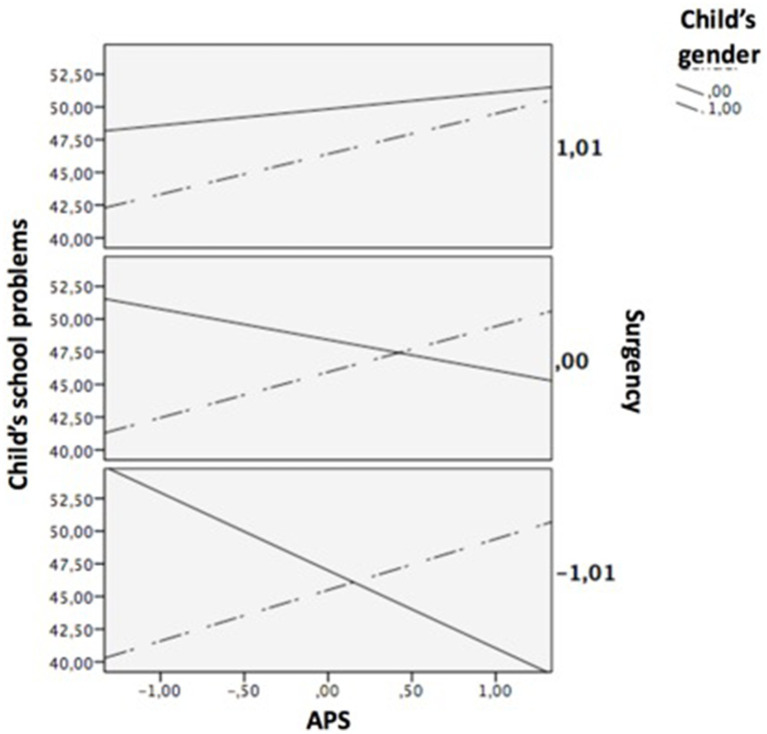
The moderation of gender in the relations between APS and child’s school problems, regarding the level of surgency.

The model predicting APS revealed 6.1% of explained variance [*F*_(4, 189)_ = 2.973, *p* = 0.021]. Father’s educational level was the only significant predictor of APS, whereas any covariates did not significantly predict the criterion (Table 4). The regression model predicting child’s school problems revealed 21% of explained variance [*F*_(14, 189)_ = 3.239, *p* = 0.000]. As expected and in line with H6 (see Table 4), the three-way interaction Surgency × Gender × APS was statistically significant (b = −3.966, *p* = 0.025). However, the three-way interaction Surgency × Gender × father’s educational level was not statistically significant (b = −1.859, *p* = 0.109), hence H6 was only partially supported. The three-way interaction Surgency × Gender × APS significantly increased the amount of explained variance by 2.3% [*F*_(1, 174)_ = 5.106, *p* = 0.025]. The conditional effects of APS × child’s gender on child’s school problems at different levels of surgency revealed that this two-way interaction was a significant positive predictor of school problems when surgency was low (b = 9.853, *p* = 0.000) and medium (b = 5.844, *p* = 0.000), but not when surgency was high (b = 1.835, *p* = 0.360). Thus, as shown in Table 5, the relation between APS and school problems was significant for girls, regardless the surgency level, whereas among boys, only when they had a low level of surgency, this relation was significant. Simple slopes analyses were computed at the mean of surgency and ± 1 SD, producing the plots in Figure 3. Johnson-Neyman technique provided additional information, showing that the value 0.653 of surgency is the one that splits different regions of signification, in such a way that the interactive effect APS × child’s gender on school problems is significant for surgency values below of 0.653 (74.1%) and is non-significant for surgency values above of 0.653 (25.9%).

**Table 4:** Testing the moderating effects of gender and surgency in the mediating effect of APS in the relation between father’s educational level and child’s school problems.

Predictors	Outcome variables					
	APS			School problems		
	*b*	SE	*t*	*b*	SE	*t*
Father’s EL	−0.147	0.052	−2.838**	−1.339	0.725	−1.847
APS				−2.389	1.266	−1.887
Gender				−4.573	3.187	−1.435
Father’s EL × gender				0.758	1.006	0.753
APS × gender				5.889	1.566	3.762***
Surgency				−2.467	1.867	−1.321
Father’s EL × surgency				1.381	0.65	2.126*
APS × surgency				3.564	1.316	2.708**
Gender × surgency				4.262	3.667	1.162
Father’s EL × gender × surgency				−1.859	1.153	−1.612
APS × gender × surgency				−3.966	1.755	−2.260*
Father’s age	0.011	0.015	0.703	−0.352	0.149	−2.363*
Father’s ES	0.025	0.08	0.316	−0.956	0.78	−1.226
Number of siblings	−0.170	0.109	−1.557	1.53	1.071	1.428
*R* ^2^	0.061			0.207		
F	2.973*			3.239***		

Each column is a regression model that predicts the criterion at the top of the column. Gender was dummy coded such that 0 = Boy and 1 = Girl. Results are based on 5,000 bootstrap samples. Father’s EL, Father’s educational level; Father’s ES, Father’s employment situation. *p < 0.05, **p < 0.01, ***p < 0.001.

**Table 5:** The conditional effect of APS on child’s school problems for girls and for boys at values of surgency.

Child’s gender	Surgency	Effect	SE	*t*	*p*-value	LLCI	ULCI
Boys	Low	−5.951	2.214	−2.687	0.008	−10.322	−1.581
	Medium	−2.349	1.259	−1.865	0.064	−4.834	0.137
	High	1.254	1.344	0.933	0.352	−1.399	3.906
Girls	Low	3.902	1.516	2.573	0.011	0.909	6.895
	Medium	3.495	0.919	3.803	0.000	1.681	5.310
	High	3.088	1.480	2.086	0.038	0.167	6.010

The results are based on 5,000 bootstrap samples and included control variable. LLCI, Lower limit confidence interval; ULCI, Upper limit confidence interval.

The overall index of moderated moderated mediation was significant (index = 0.583, SE = 0.337, 95% CI [0.003, 1.311]), supporting H7. More specifically, results revealed that the moderated mediation effect was significant in the conditions of low and medium levels of surgency (index = −1.447, SE = 0.634, 95% CI [−2.835, −0.373] and index = −0.858, SE = 0.375, 95% CI [−1.696, −0.231], respectively), but not in the condition of high level of surgency (index = −0.270, SE = 0.334, 95% CI [−1.023, 0.298]). In the condition of low level of surgency, the effect of father’s educational level on child’s school problems was significant and positively mediated by APS for boys (index = 0.874, SE = 0.420, 95% CI [0.162, 1.809]), but significant and negatively mediated for girls (index = −0.573, SE = 0.348, 95% CI [−1.369, −0.011]). In the condition of medium level of surgency, the effect of father’s educational level on child’s school problems was positive and non-significantly mediated by APS for boys (index = 0.345, SE = 0.219, 95% CI [−0.016, 0.851]), but significant and negatively mediated for girls (index = −0.513, SE = 0.240, 95% CI [−1.041, −0.132]). Lastly, although the index of moderated mediation was not significant in the condition of high level of surgency, it is interesting to note that the effect of father’s educational level on child’s school problems was negative and non-significantly mediated by APS for boys (index = −0.184, SE = 0.215, 95% CI [−0.634, 0.213]), and also negative but significantly mediated for girls (index = −0.454, SE = 0.301, 95% CI [−1.155, −0.008]).

## 6 Discussion

Our research aimed to test an underlying mechanism of the relation between fathers’ educational level and children’s school problems. Overall, our results when analyzed together firstly suggest that a low father’s educational level leads to a high level of overprotective behaviors (APS). Secondly, only among girls, these overprotective behaviors increase the child’s school problems and only among girls, the indirect way by APS accounts for the adverse effects of a low educational level of father on girls’ school performance. Additionally, this occurs regardless of the level of surgency of girls. Thirdly, among boys, the relation between paternal factors (educational level and APS) are different depending on the surgency level. Thus, when the level of boy’s surgency is medium or high, there are no direct or indirect effects by APS of the father’s educational level on the child’s school problems. Among boys with low levels of surgency, a low educational level of the father is directly associated with his child’s school problems. However, given that among these boys with low levels of surgency, overprotective behaviors (APS) of fathers protect their boys of school problems, the indirect effect of fathers’ educational levels on child’s school problems by APS was positive; in other words, certain overprotective behaviors of fathers as APS minimize the risk of a low educational level of fathers on the development of school problems for boys with low levels of surgency (or high levels of shyness).

Findings of the current study support the importance of fathers in children’s development, specifically in their school skills, as it has been shown in the literature (Cabrera et al., 2000; Lamb, 2010; Ramchandani and Psychogiou, 2009; Sarkadi et al., 2008). Our results also support the studies that have detected relations between fathers’ educational attainment and different children’s academic skills (D’Urso and Symonds, 2022; Sarkadi et al., 2008). Some arguments have been alluded to explain these results: parents with higher educational level could feel more empowered and so more capable to intervene and influence their children’s academic skills (Veiga et al., 2016); parents with higher educational level use more elaborate language and create environment that facilitate learning, which affects children’s academic performance (Hoff, 2003; Williams, 1980); or parents with higher educational levels have higher expectations for their children education and show interest in their children’s academic performances which will be achieved with academic outcomes (Alexander et al., 1994; Good and Brophy, 1990). In addition, our findings confirm that parental involvement is a key mediator of the association between parental educational level and their children’s school problems (Davis-Kean, 2005; Pettit et al., 2009). Moreover, our results empirically support the directional influence and the theoretical model, proposed by Harding et al. (2015), which declares that with additional years of education, parents increased access to human (e.g., problem-solving ability), cultural (e.g., network connections), and social (e.g., high-achieving role models) capital, which shape their parenting behaviors and, in turn, their children’s school outcomes.

In our view, this study also extends research on relations between father’s educational level, parenting and school adjustment in several ways. First, we have examined the mediating role of a parental practice that is quite widespread today and that previous research, carried out mainly with mothers and at other ages, has pointed out that it can be adverse for the development of their children, depending on the child’s age. Overparenting behaviors as APS are a common practice in Western countries (LeMoyne and Buchanan, 2011). APS implies being highly attentive to potential risk and problems affecting the child, and a tendency to act before problems arise or have been perceived as such by the children (Scharf et al., 2017). As fathers are more involved in fulfilling the needs of their children, APS means paternal care and support for their children (Leung and Shek, 2019). Although, these behaviors appear to be beneficial in early stages of development such as the preschool years (Schiffrin et al., 2015), in adolescents and young adults, it has been shown to be a harmful practice, given that it increases dependency on others (Odenweller et al., 2014), and decrease levels of sense of competence and control (Hong and Cui, 2020). Present study’s findings suggest that in early middle childhood—a stage in which studies on these relationships have been rare or absent to our knowledge—a higher educational level protects fathers to manifest APS, while a lower educational level enhances APS behaviors and, in turn, increases the problems of their children in schools, only for the case of girls. It seems that, as young as 7 or 8 years old, girls need parents who are sensitive to changes in their development and allow them to cope independently with problems that they would be able to solve on their own and that APS behaviors can be considered intrusive at these ages. These findings are consistent with self-determination theory (Ryan and Deci, 2000).

However, for boys, the relations between fathers’ overprotective behaviors with their children’s school problems depend on the boys’ level of surgency, in such a way that as the surgency decreases (boys are more inhibited or shy), these paternal behaviors seem to protect them from developing school problems. From early childhood to adolescence, there is increasing evidence that suggests that a low level of surgency (or a high level of shyness) may be less socially acceptable for boys than for girls, probably due to this behavior in boys seems to violate Western gender expectations that characterize boys as more active, assertive and dominant (Doey et al., 2014; Rubin and Coplan, 2004). Thus, shy boys tend to experience more peer rejection, more anxiety, stress, emotional difficulties and perceive themselves to be less physically and cognitively competent than shy girls (Coplan et al., 2004; Howarth et al., 2013; Nelson et al., 2005). As a result, the consequences of shyness are more problematic for boys, being at a greater disadvantage in their social and academic settings, than for girls (Butt et al., 2019).

Furthermore, parents who perceive that their shy boys have difficulties, could increase their overprotective behaviors, interfering in boys’ activities and anticipating and solving child’s problems (Hastings et al., 2010; Rubin et al., 1999) in order to support their sons. In fact, shyness appears to predict protection and related parenting behaviors (Edwards et al., 2010; Kiel and Buss, 2012). On the other hand, previous studies have indicated that boys and girls may differ in their reactions to different parenting styles (Lengua, 2008; Pitzer et al., 2011). In this way, some authors speculate that girls are more sensitive to the pressure and demands, perceiving overprotective behaviors more as intrusion and control than as support (Pettit et al., 2001).

Probably during the transition to elementary school, parents’ overprotective behaviors could have helped to cope with the new educational challenges that children have had to face (Leung et al., 2021; Serbin et al., 2013). However, the earlier development of self-regulation abilities in girls (Hosseini-Kamkar and Morton, 2014; Matthews et al., 2009; Weis et al., 2013) could explain that a bit later, at 7 or 8 years old, girls need a higher level of autonomy. However, shy children, for whom new school challenges often cause greater stress and anxiety (Hudson and Rapee, 2004), could need the withdrawal of overprotective paternal behaviors to occur more gradually and slowly than in girls, as boys improve their self-regulation skills.

### 6.1 Limitations and directions

The current study provided important knowledge to advance our understanding of the direct and indirect influence of paternal factors on children’s problems at school. However, our research still has some limitations that point out future research directions. First, we used a community sample of children from Cádiz (southern Spain); thus, our profile of temperamentally shy boys may not capture the extreme temperamental trait and these findings may not be generalizable to other groups and other geographical regions. Future studies are needed to replicate these findings in more diverse samples of children. Second, the current study included different informants (fathers reported their overparenting behaviors, mother and father jointly informed about children’s surgency and teachers reported children’s school problems) and the measuring instruments selected are widely used. However, the use of parent-reported data as our measure of surgency could be restricted to the contexts that parents observe their children, and their responses may have been biased by social desirability. Future work should improve reliability using additional informants for each measure and using complementary other methods as direct observation or physiological measures. Third, the PROCESS macro in SPSS was used to test the proposed mediation–moderation model which was based on previous literature and theory. However, and although data was collected at three time points, the used cross-sectional design does prevents us determining the direction of causality among the variables. Future studies using longitudinal designs are needed to confirm these findings. Fourth, our initial interest focused on the role of overparenting as mediator of the relation between fathers’ educational level and children’s school problems and the moderating role of gender and surgency. Nevertheless, future studies could explore alternative explanations concerning how a low educational level of the father can lead to the school problems of his children.

### 6.2 Theoretical contributions

Self-determination theory (Ryan and Deci, 2000) has been shown as a suitable psychological framework to assess the influence of overparenting behaviors on children’s school problems. Considering that the basic human need for autonomy requires to be satisfied in order to assure an individual’s wellbeing, the theory explicitly supports that overparenting behaviors could threaten their children’s school adjustment preventing the satisfaction of their needs for autonomy. The current research adds that the consequences of this parental style depend on variables such as gender and age, so that boys and girls may differ in their need for personal autonomy based on their rate of development, challenges, and school transitions.

On the other hand, our findings add empirical evidence to the Environmental Sensitivity framework (elaboration on the various theories as environmental susceptibility or diathesis-stress; Ellis et al., 2011; Pluess, 2015), showing that the effects of two environmental factors (paternal educational level and overprotection) on school maladjustment are moderated by individual variables, such as gender and surgency. Moreover, the current study is in line with others that show that environmental sensitivity is better conceptualized as multidimensional and better understood when it is studied employing a person-centered approach (Markovitch et al., 2023; Moran et al., 2017). In this sense, our findings suggest that it is the combination of one’s tendency to shyness and low extraversion with gender that possibly has a unique role in environmental sensitivity rather than each of these individual characteristics separately. Understanding the complex individual differences in environmental sensitivity requires more research by various analytic strategies.

### 6.3 Practical implications

Given the noted relevance of adjusting to elementary school to the development of later socioemotional and academic skills, and the growing trend of parents in western countries to overprotect their children, the present study poses some practical implications. First, for teachers and parents, our findings highlight the importance of an early identification of children who display high levels of shyness, especially among boys, in order to reinforce the support so that they can overcome the challenges and threats at school, gradually promoting autonomy. Moreover, the fact that girls appear to be more sensitive to adverse effects of overprotective behaviors of their fathers, programs guiding parents to handle these behaviors which play a key role in their daughters’ school problems. Further, these programs are especially crucial for those fathers with low educational attainment. These probably well-intended fathers should be aware that helping their daughters too much to solve their problems at school, when they could deal with them, could have harmful consequences for their progress in educational settings.

In this sense, findings raise implications for educational settings since it can support programs aimed at parents with a low educational level and at risk of displaying overparenting behaviors, considering children’s characteristics.

## Data Availability

The database presented in this study can be found in online repositories. The name of the repository and accession number can be found at the Zenodo Repository https://doi.org/10.5281/zenodo.10732833.
